# How best to interpret measures of levels of oxygen in tissues to make them effective clinical tools for care of patients with cancer and other oxygen‐dependent pathologies

**DOI:** 10.14814/phy2.14541

**Published:** 2020-08-12

**Authors:** Harold M. Swartz, Ann Barry Flood, Philip E. Schaner, Howard Halpern, Benjamin B. Williams, Brian W. Pogue, Bernard Gallez, Peter Vaupel

**Affiliations:** ^1^ Department of Radiology Dartmouth Medical School Hanover NH USA; ^2^ Department of Medicine Section of Radiation Oncology Dartmouth‐Hitchcock Medical Center Lebanon NH USA; ^3^ Thayer School of Engineering Dartmouth College Hanover NH USA; ^4^ Department Radiation and Cellular Oncology University of Chicago Chicago IL USA; ^5^ Department of Surgery Dartmouth‐Hitchcock Medical Center Lebanon NH USA; ^6^ Louvain Drug Research Institute Université catholique de Louvain Brussels Belgium; ^7^ Department Radiation Oncology University Medical Center University of Freiburg Freiburg Germany; ^8^ German Cancer Center Consortium (DKTK) Partner Site Freiburg German Cancer Research Center (DKFZ) Heidelberg Germany

**Keywords:** clinical measures of oxygen, oxygen in tissues, partial pressure

## Abstract

It is well understood that the level of molecular oxygen (O_2_) in tissue is a very important factor impacting both physiology and pathological processes as well as responsiveness to some treatments. Data on O_2_ in tissue could be effectively utilized to enhance precision medicine. However, the nature of the data that can be obtained using existing clinically applicable techniques is often misunderstood, and this can confound the effective use of the information. Attempts to make clinical measurements of O_2_ in tissues will inevitably provide data that are aggregated over time and space and therefore will not fully represent the inherent heterogeneity of O_2_ in tissues. Additionally, the nature of existing techniques to measure O_2_ may result in uneven sampling of the volume of interest and therefore may not provide accurate information on the “average” O_2_ in the measured volume. By recognizing the potential limitations of the O_2_ measurements, one can focus on the important and useful information that can be obtained from these techniques. The most valuable clinical characterizations of oxygen are likely to be derived from a series of measurements that provide data about factors that can change levels of O_2_, which then can be exploited both diagnostically and therapeutically. The clinical utility of such data ultimately needs to be verified by careful studies of outcomes related to the measured changes in levels of O_2_.

## 1 INTRODUCTION

The overall goal of this review is to facilitate clinically effective use of measurements of molecular oxygen (O_2_) in tissues with the explicit intent of improving clinical care, that is, improving the accuracy and effectiveness of diagnoses, treatments, and prognoses for individual patients. This review focusses especially on improving personalized medicine and outcomes of care, by carefully considering the basis and validity of clinical measurements of O_2_ in tissues and how those measurements can be used to advance diagnosis and therapy. While measurements of O_2_ in tissues have been recognized as an important factor in the clinical evaluation and treatment of many diseases, especially cancer (Busk, Overgaard, & Horsman, 2020), and pathologies involving ischemia (such as in peripheral vascular disease and wound healing), insufficient attention often has been paid to the meaning of the values that have been obtained. (Note: this review is derived, in part, from a series of recent papers on this topic; Flood et al., 2020; Swartz, Flood, et al., 2020; Swartz, Vaupel, et al., 2020.)

Instead, all too often, when a measurement technique has indicated that the level of O_2_ in a given tissue is “X,” that is, is some specific quantitative number for the O_2_ in the tissue, researchers, and clinicians alike assume that “X” is a reliable, accurate representation of the “true” oxygenation status of the tissue. This approach ignores the complexity and dynamics of O_2_ in living biological systems. The reality is that any O_2_ measurement has been taken at only one point in time of a distribution in the subvolume that was interrogated by the method, while the O_2_ is in fact varying with time and across space in the tissue and is unlikely to be uniform in the volume that is being interrogated.

In this review we focus on the biological/clinical meaningfulness of O_2_ measurements made in living organisms, while recognizing that tissue O_2_ is in constant flux. We emphasize that, to obtain maximum clinical utility of the measurements, it is necessary to consider the goal of the measurements and the limitations of the data that are obtained. We particularly focus on the clinical value of making repeated measurements of O_2_, especially in association with strategies/events that potentially change O_2_ levels.

## 2 WHAT ARE OXYGEN LEVELS IN TISSUES?

### 2.1 Physical concepts and terminology for reporting on oxygen levels in tissues

The level of molecular oxygen, that is, O_2,_ is usually reported as partial pressure of oxygen (pO_2_) or concentration of oxygen ([O_2_] or cO_2_). These terms have physically rigorous meanings that can usefully be extended to describe gases (such as O_2_) that are dissolved in liquids or solids, including tissues. Partial pressure is the pressure exerted by oxygen in a mixture of gases, while concentration is the content of oxygen in the gas mixture or solid. Partial pressure is commonly expressed in mmHg, and these units are sometimes referred to as torr or kPa (SI unit used in the EU), while concentration of O_2_ is commonly expressed in mL of O_2_ per 100 ml, for example, in blood.

However, the solubility of oxygen varies greatly in different media (Bennett, Swartz, Brown, & Koenig, 1987; Jordan et al., 2013) and this affects the relationship between pO_2_ and [O_2_]. The transport of O_2_ across lipid membranes is known to depend on both diffusion and solubility in the bilayer, and to be affected by changes in the physical state and by the lipid composition, especially the content of cholesterol and unsaturated fatty acids. For example, because O_2_ partitions preferentially into lipophilic media, such as membranes, the solubility of O_2_ in membranes is about four times greater than in aqueous solutions (Möller et al., 2016). This difference has significant consequences for physical and chemical interactions involving molecular oxygen in biological systems because these interactions depend on the number of oxygen molecules that are present and their rate of diffusion.

Does it matter clinically to know whether the technique is reporting [O_2_] or pO_2_? While these measures are not identical, there is a known relationship between them. According to the ideal gas law, pO_2_ is directly proportional to concentration, assuming the volume and temperature are constant, that is,

PV=nRT

where P = pressure (pO_2_); V = volume; *N* = amount of substance [O_2_]; R = ideal gas constant; T = temperature.

It is less straightforward in biological systems. If the solubility of oxygen in each component of tissue is known (and this is not always fairly readily derived experimentally) and pO_2_ can be measured, then it is relatively straightforward to calculate [O_2_]. Conversely, if the component in which [O_2_] is measured and the solubility of O_2_ in that compartment is sufficiently known, then it should be feasible to determine pO_2_. However, it often is not feasible to measure these parameters readily.

Because of the biological complexities in assessing O_2_ in tissues, each reported measure of O_2_ level in a tissue can be better considered as an average value. *Average* is used here in its more colloquial usage rather than as a statistically defined term, because different techniques output their measurement of O_2_ using differing methods pertinent to that technique. Each technique gathers information from a particular volume of tissue (irrespective of whether that volume is well characterized), which we refer to hereafter as the “interrogated volume.” The sampling of data within the interrogated volume is then used to produce a measure based on a sort of average O_2_ within that volume. Characterizing that “average measure” is made difficult both by the imprecision of knowing the exact volume queried, but also because the detection of O_2_ in that volume may be affected by factors such as its distance from the detector or, for optical techniques, different rates of scattering (that also may not be well characterized). Hence, we conclude that it is important to bear in mind that the measurements of O_2_ in vivo are fundamentally based on a sort of “average” within the interrogated volume.

Because of all of these challenges in obtaining precise measurements of relevant parameters necessary to assess whether the data obtained by any technique is truly measuring either pO_2_ or [O_2_] and because of the imprecisions of knowing the volume being assessed and the tissues within that volume, it is more realistic to acknowledge the complexity of these issues for in vivo measurements in tissues by using a less precise term for measures of molecular oxygen in tissues such as “O_2_ levels,” which is the convention we follow in this review. We also argue that, while it is important to recognize the biological imprecisions in these measures, there are still many clinically viable uses of this information (such as assessing change in O_2_ levels).

Note too, within this paper, while focusing on the uncertainties due to sampling issues of each technique and variations due to biological factors, we are not taking into consideration further uncertainties due to inevitable instrumental noise, variations in the placement of the detector, etc.

### 2.2 Expected levels of O_2_ in tissues

Table 1 presents some illustrative data on the O_2_ levels in various tissues, both in normal states and as altered by some diseases. (These measures are presented here as reported in the literature.) The first column presents the median pO_2_ obtained, using the Eppendorf electrode (or comparable polarographic techniques) to measure O_2_ levels in patients; also presented are two other indications of O_2_ levels: the hypoxic fraction (the percentage of measurements in a given type of tissue that is below a defined “hypoxic level,” in this case 2.5 mmHg) and the range of pO_2_ values found experimentally. These data illustrate both the variation in median O_2_ levels between types of tissues and how they may vary with physiology or disease. For example, (intertissue) in general the median O_2_ levels are lower in skeletal muscle and heart compared to the spleen; (intratissue) the median O_2_ in skeletal muscle at rest is higher than with exercise while, in contrast, there is almost no variation between the normal spleen and with Hodgkin's disease. The data in Column 4 illustrate the wide range that any given measurement can have in the “same” type of tissue, that is, almost all tissues range from ~0 to ~100 mmHg, even when their median value is quite different (see again spleen vs. bone). However, these very high values in the upper range may include experimental artifacts due to the measurement being taken within or very close to an arteriole, for example, a vessel feeding the microcirculatory bed.

**Table 1:** Oxygenation status of organs/tissues

Organ/tissue	Median pO_2_ (mmHg)	HF 2.5 (%)	pO_2_ range (mmHg)	References
Kidney				
Cortex	45–50	1–2	1–97^#^	Günther, Aumüller, Kunke, Vaupel, and Thews (1974)
Outer medulla	38	2–5	2–96	Same
Inner medulla	11	9–11	0–32	Same
Liver	25–30	1–2	1–96	Kallinowski and Buhr (1995a)
Pancreas	57	2	1–95	Koong et al. (2000)
Spleen				
Normal	68	1–2	2–96	Vaupel, Wendling, Thomé, and Fischer (1977)
In hypersplenism	69	2	2–97	Wendling, Vaupel, Fischer, and Brünner (1977)
Hodgkin's disease	67	2	2–96	Same
Myocardium				
Subepicardial	18–26	1	1–96	Winbury, Howe, and Weiss (1971)
Subendocardial	10–17	n.a.	1–94	Moss (1968) (for both)
Mucosa				
Oral	52	1	1–96	Kallinowski and Buhr (1995a)
Rectal	51	n.a.	1–95	Same
Large bowel	55	n.a.	1–95	Same
Breast				
Normal	65	0	10–96	Vaupel, Schlenger, Knoop, and Höckel (1991), Vaupel and Harrison (2004)
Fibrocystic disease	67	0	5–98	Same
Prostate	26	4	1–96	Vaupel and Kelleher (2013)
Uterine cervix	41	8	1–97	Höckel, Schlenger, Knoop, and Vaupel (1991)
Subcutis	52	0	0–96	Same
Bone				
Cortical	32	3	0–96	Spencer et al. (2014)
Hematopoietic marrow	22	1	0–95	Same
Adipose marrow	26	2	0–95	Same
Skeletal muscle				
Resting	27–32	0–2	0–96	Landgraf and Ehrly (1980)
Exercise	10	5–10	0–96	Jung, Kessler, Pindur, Sternitzky, and Franke (1999)
Hypovolemic shock	4	40	0–40	Harrison and Vaupel (2014)
PAOD	6–7	~30	0–90	Landgraf, Schulte‐Huermann, Vallbracht, and Ehrly (1994)
Skin				
Thermoneutral conditions	25–35	n.a.	40–70	Carreau et al. (2011)
Critical limb ischemia	5–8	18	0–96	Harrison and Vaupel (2014)
Limbs, venous disease	15	n.a.	40–65	Clyne, Ramsden, Chant, and Webster (1985)
Brain				
Gray matter	28	1	1–96	Vaupel (1994)
White matter	10–15	n.a.	n.a.	Same
Retina	~20	n.a.	0–70	Hogeboom van Buggenum, van der Heijde, Tangelder, and Reichert‐Thoen (1996), Linsenmeier and Zhang (2017)
White adipose tissue				
Nonobese	56	n.a.	40–74	Pasarica et al. (2009), Hodson (2014)
Obese	47	n.a.	29–63	Lempesis, van Meijel, Manolopoulos, and Goossens (2020)

Abbreviations: #: arterial; HF2.5: hypoxic fraction ≡ fraction of pO_2_ values ≤2.5 mmHg; n.a.: information not available; PAOD: peripheral arterial occlusive disease.

Table 2 presents the same types of information for a more detailed analysis of changes in an important pathology, cancer, where O_2_ levels have been an especially important focus for informing clinical treatment and prognosis. To give the reader a sense of how well supported the numbers are, the data in Table 2 have been ordered by the number of patients included in each row.

**Table 2:** Pretherapeutic oxygenation status of human tumors

Tumor type (ordered by no. of patients)	No. of patients	Median pO_2_ (mmHg)		HF 2.5 (%)	pO_2_ range (mmHg)	References
Cervix cancer	730	9–10	28		0–88	Vaupel et al. (2007)
Head and neck cancer	592	10	21		0–90	Vaupel (2009)
Prostate cancer	438	7	26		0–95	Vaupel (2011)
Soft tissue sarcoma	283	14	13		0–96	(data synopses)
Breast cancer	212	10	30		0–95	*These 3 ref. apply to all*
Glioblastoma	104	13	26		0–50	*Above the line*
Vulvar cancer	54	11	25		0–92	Vaupel, Thews, Mayer, Höckel, and Höckel (2002), Vaupel, Mayer, and Höckel (2006), Stone et al. (2005)
Rectal cancer	29	25	n.a.		0–92	Kallinowski and Buhr (1995a), Mattern, Kallinowski, Herfarth, and Volm (1996)
Lung cancer	26	16	13		0–95	Falk, Ward, and Bleehen (1992), Le et al. (2006)
Malignant melanoma (metastatic)	18	12	5		0–96	Lartigau et al. (1997)
Non‐Hodgkin's lymphoma	8	18	36		0–92	Powell et al. (1999)
Pancreas cancer	8	2	59		0–91	Koong et al. (2000), Graffman, Bjork, Ederoth, and Ihse (2001)
Brain metastases	5	10	26		0–87	Rampling, Cruickshank, Lewis, Fitzsimmons, and Workman (1994)
Liver metastases	4	6	n.a.		0–90	Kallinowski and Buhr (1995a, 1995b)
Renal cell carcinoma	3	10	n.a.		0–90	Lawrentschuk et al. (2005)
Gall bladder cancer	1	4	n.a.		0–10	Graffman et al. (2001)
Bile duct cancer	1	8	n.a.		0–15	Graffman et al. (2001)

Abbreviations: HF2.5: hypoxic fraction ≡ fraction of pO_2_ values ≤2.5 mmHg; n.a.: information not available.

Of interest here, all seven cancer types with at least 50 patients studied have a fairly consistent and fairly hypoxic median O_2_ level (~10 mmHg). Similarly, all but soft tissue sarcoma have a similar hypoxic fraction, ~25%; (sarcoma appears to be about half that). Glioma appears to be an outlier on the range; glioma had no patient whose O_2_ level was above 50 while all others (as was true for the tissues in Table 1) have at least one measurement in the upper 80s or 90s. These occasional high readings are not surprising since it is plausible that, randomly, some readings will have been obtained in or very near to arterioles/feeding microvessels. In contrast to the first seven cancers, the 10 types of cancers with fewer than 30 patients appear to be more varied in their O_2_ levels, but this is possibly due to being based on few patients.

Nevertheless, even though (as noted elsewhere in this review), the data presented are not unconditionally “absolute values” of O_2_ levels (as they are sometimes referred to; e.g., Koch, 2002; Macnab, Gagnon, Gagnon, Minchinton, & Fry, 2003). Nevertheless (as argued in this review), they can provide very useful data as long as their limitations are recognized by researchers and clinicians.

## 3 HETEROGENEITY OF DISTRIBUTIONS OF LEVELS OF OXYGEN IN TISSUES: EXAMPLES AND CAUSES

**Figure 1:**
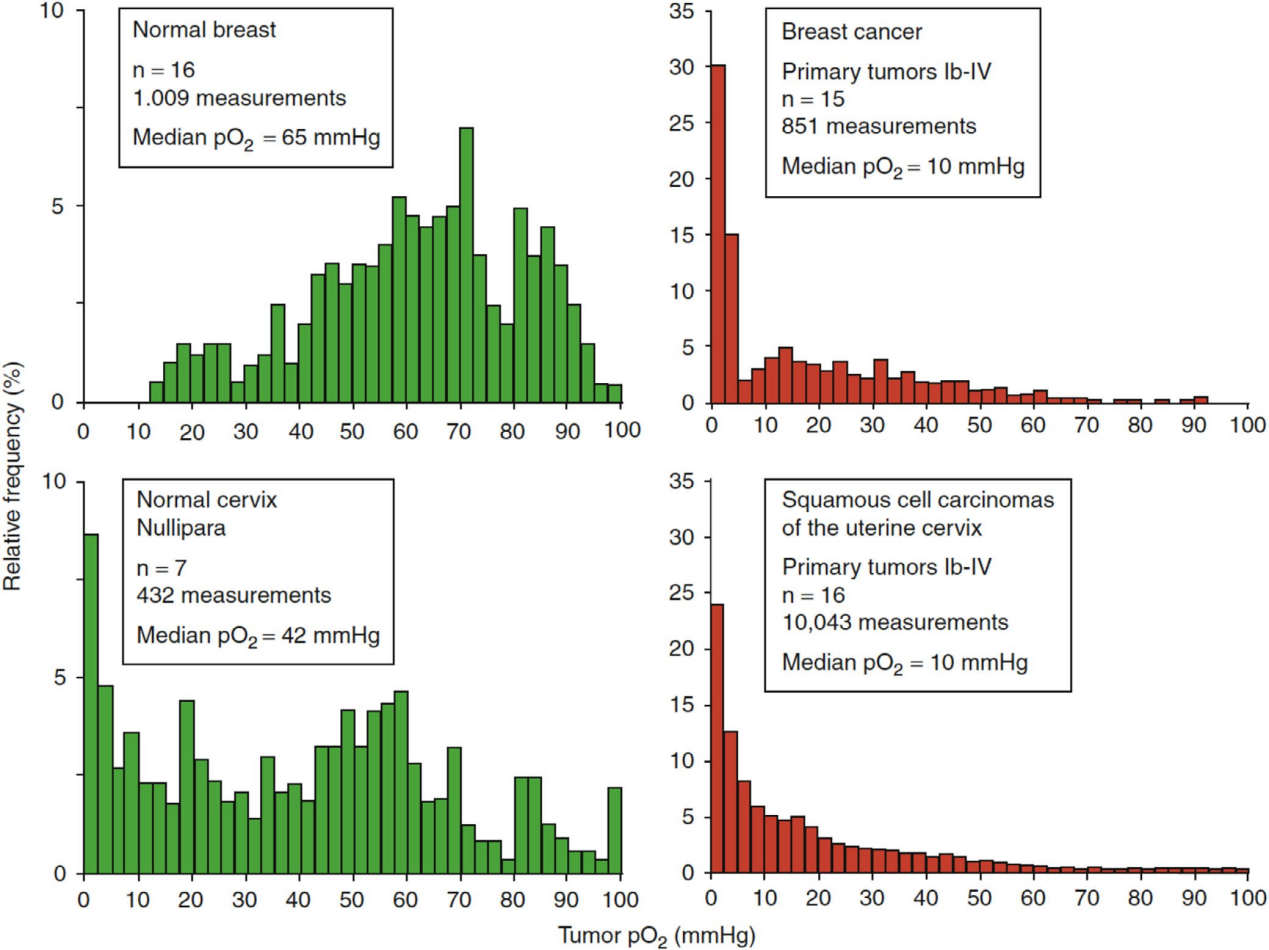
Distributions of multiple O_2_ levels made in patients with normal versus malignant tissue: Breast and cervix. (Figure adapted from Vaupel & Mayer, 2017b, p. 3,343)

**Figure 2:**
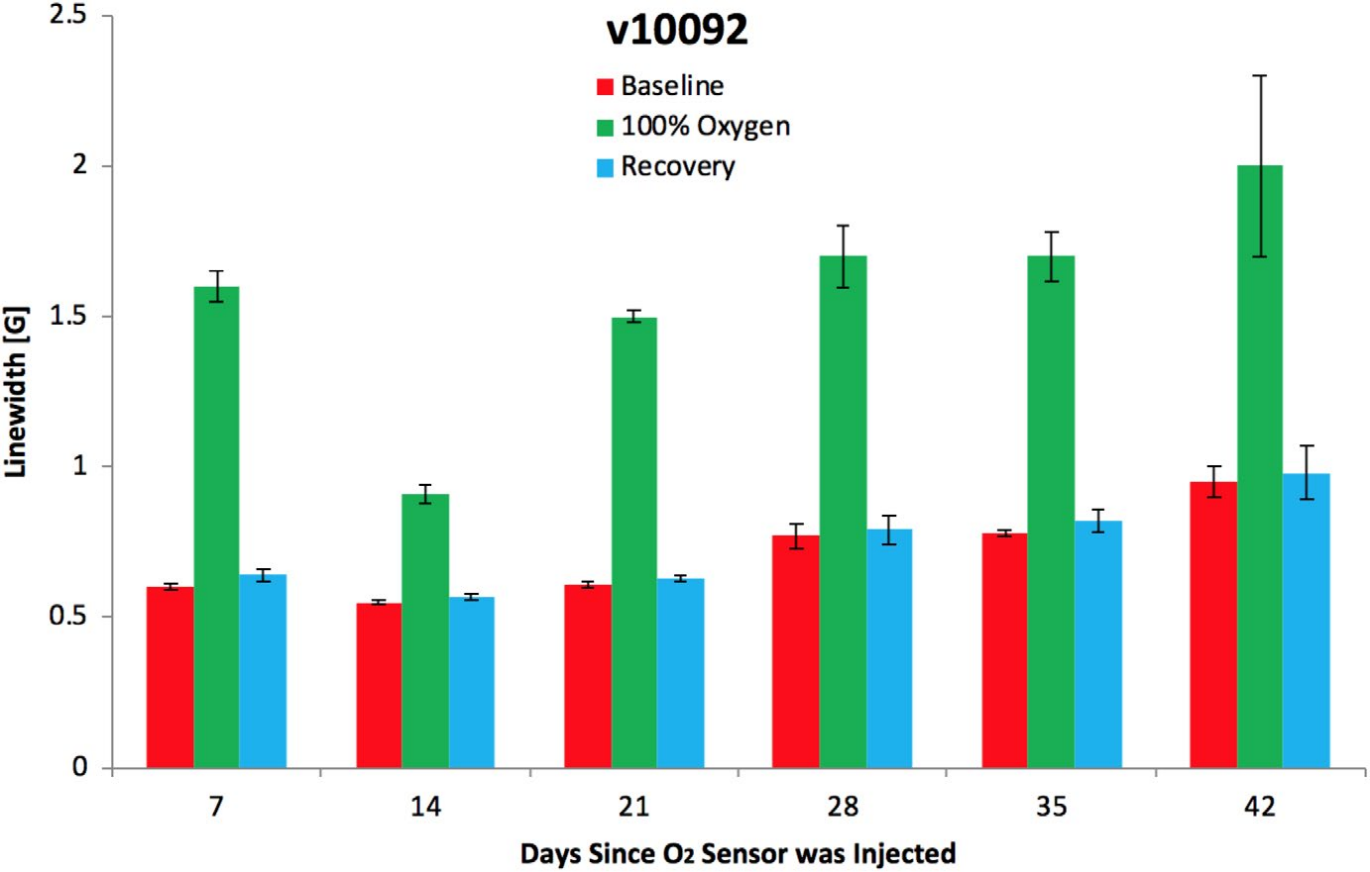
Repeated O_2_ level measurements^A^ during each measurement session and over 42 days: Breast cancer patient measured in skin and superficial breast tissue within the radiation field during a course of radiation therapy. (Figure adapted from Flood et al., 2020, p. 164.) ^A^For Carlo Erba ink, EPR line width increases with increasing O_2_ level, but the relationship is nonlinear and can be impacted by several factors. Therefore, the data are given here as line width

Heterogeneity of distributions of oxygen values in the tissues of interest exists over many dimensions, including time and space, and over a wide range of scales (Harrison & Vaupel, 2014). Tables 1 and 2 focus on intertissue and intratissue variation in overall levels of O_2_. Figures 1 and 2 illustrate heterogeneity using more refined data points to illustrate the skewed nature of the data, particularly for malignancies. The data in Figure 1 are based on multiple measurements made in a series of patients using a computerized polarographic microsensor technique which enables direct assessment of the O_2_ levels with an O_2_‐sensitive needle electrode (subject to the limitations of providing true absolute values as discussed elsewhere in the paper). Oxygen was measured along several electrode tracks in each individual during a given measurement session (from near the tumor surface up to tissue depths of ~ 30 mm in breast cancers and in cancers of the uterine cervix). Each row in Figure 1 presents a type of tissue (breast and uterine cervix) with comparisons of O_2_ levels made in normal tissue (green) versus malignancies (prior to treatment) of these organs (red). The summary measures, median pO_2_ values (parallel to data reported in Tables 1 and 2), are included in the text boxes.

Note that the data in Figure 1 are not normally distributed; (the distribution of O_2_ levels made in normal breast tissue is the closest approximation to a normal distribution). Comparing the two distributions for malignancies, we see that breast cancer is more highly skewed than cervical cancer, although they have the same median. Thus, using a single measure such as the median could overlook potentially important clinical information.

Similarly, comparing the distributions for the normal tissues, the normal cervix had a substantial number of O_2_ measurements within a hypoxic range defined as ≤10 mmHg, while there were no hypoxic measurements in the normal breast. While Figure 1 does not differentiate the measurements made per patient, it illustrates why several authors report the hypoxic fraction when trying to capture a meaningful overall number to characterize a tissue. Finally, these data underscore why it is important to understand what is well captured by a given measure of O_2_ level—and what is missed or obscured.

Another example of heterogeneity is presented in Figure 2. These O_2_ measurements were taken in a breast cancer patient using EPR oximetry with Carlo Erba ink as the O_2_ sensor (Flood et al., 2020; Jeong et al., 2019). The data are presented as line widths, because the EPR oximetry technique using India Ink as a sensor undoubtably gathers data from volumes too large to have homogenous oxygen levels; however, because this sensor remains in the same place in the tissue, it still can provide very useful indications of changes over time and/or the impact of interventions such as breathing enriched oxygen. The data were taken in 30‐min sessions, during which EPR spectra were collected continuously, but the period was divided into three 10‐min periods, differing by the gas mixture the patient was breathing: room air (red: baseline), 100% O_2_ delivered by a nonrebreather mask (green), followed by again breathing room air (blue: recovery). The sessions were repeated approximately weekly throughout the period of radiation therapy (thus mimicking a clinical course of radiation therapy, although there was no attempt to impact therapy in this study).

The data for this particular patient illustrate that the O_2_ levels responded to the patient's breathing an hyperoxic gas mixture and then returned rapidly to the baseline level after the 10‐min period. In this patient, there appeared to be some variation across the weeks of treatment, with the final levels for each period (baseline, 100% O_2_, and recovery) all being slightly higher than at the beginning of radiation therapy (based on nonoverlapping standard deviations of the first and last measurements).

### 3.1 Causes of heterogeneity in normal tissues

There are spatial variances in oxygen levels in normal tissues due to the longitudinal gradient in oxygen as the blood passes through the microcirculatory bed (decreasing from the arterial inlet to the outlet of the microvessels; Erickson et al., 2003). After the oxygen leaves the microvascular networks, the partial pressure of O_2_ decreases due to radial gradients, that is, O_2_ diffuses through the tissues as it gets further from the vessels, due to O_2_ being consumed by the cells. As a result, there are variations in O_2_ levels from cell to cell (according to their distance from the microvessel). Within the cells O_2_ decreases in a microspatially complex manner as it is intracellularly consumed, with most of the consumption occurring in the mitochondria. There is growing evidence that diffusion of O_2_ into the cell may be constrained, that is, that O_2_ does not freely and rapidly flow into cells across the membrane, and therefore there are gradients from outside to inside of cells (Khan et al., 2003; Kurokawa et al., 2015; Pias, 2020).

These variations of O_2_ levels, that is, gradients between and within cells, cannot currently be measured. As detailed below in discussing temporal variations, even if such measurements of spatial heterogeneity could be made, they would still be inadequate to understand the full complexity of heterogeneity of O_2_. For example, in some normal tissues, there is additional significant macroscopic heterogeneity of O_2_ over space because of their physiology as a consequence of substantial differences in vascularity, blood flow, and oxygen consumption (e.g., macroscopic heterogeneity between gray and white matter of the brain, between subepicardial and subendocardial layers of the myocardium, and between renal cortex and renal inner medulla).

There also are temporal changes in O_2_ levels in normal tissues (Griffith, 1996). Moreover, the supply of O_2_ can vary periodically due to rhythmic changes in microcirculatory blood flow which is reflected at all levels from the inflow arteries to the microcirculation. Finally, within the microcirculation there are variations in microvascular flow due to regional regulation in response to varying metabolic demands (Kimura et al., 1996). Important differences in O_2_ solubility across tissues, affecting the relationship between pO_2_ and [O_2_], were discussed earlier.

### 3.2 Impact of pathology on heterogeneity of O_2_ levels

The presence of pathology often significantly increases the amount and extent of oxygen heterogeneity both spatially and temporally (Vaupel & Harrison, 2004; Vaupel & Mayer, 2016). The presence of pathology often impacts the structure/morphology of the vessels. In tumors there often is a significant amount of neoangiogenesis which results in much less ordered and less functional blood vessels (Busk et al., 2020). The resulting vessels are much less efficient in delivering blood and also tend to be much more prone to leak. Leakage from these vessels can cause increases in the interstitial pressure, which can reduce the effectiveness of the microcirculation due to reduction of the perfusion pressure within the tumor capillaries (Fukumura, Duda, Munn, & Jain, 2010).

Pathological changes also can result in altered consumption of O_2_. In malignant tumors O_2_ consumption is likely to decrease due to poor oxygen delivery and/or because of a switch to glycolysis due to metabolic reprogramming (i.e., the Warburg effect) as a consequence of HIF‐1α overexpression, upregulation of oncogenes, downregulation of suppressor genes, and activation of certain signaling pathways (Vaupel & Multhoff, 2020; Vaupel, Schmidberger, & Mayer, 2019). Pathology can also impact the integrity of the blood vessels. For example, tumor growth may physically impinge on the integrity of the blood vessels, and the metabolic abnormalities in diabetes can impact the structure of blood vessels (causing either microangiopathy and/or macroangiopathy). The results of these processes can produce very significant local variations in the availability of fully functional vascular structures, resulting in locally hypoxic regions.

Pathology also can impact temporal changes of oxygen and the response to treatment. The presence of pathology, especially cancer (e.g., acute and cycling hypoxia in cancers) and peripheral vascular disease, can result in significantly greater variability in O_2_ levels (Braun, Lanzen, & Dewhirst, 1999). These include short‐term changes, especially associated with the structural abnormalities of the microcirculation resulting in increased local variability in flow, and long‐term changes that develop over time, such as those due to disease progression and responses to therapy (Baudelet et al., 2004; Kimura et al., 1996; Konerding, Fait, & Gaumann, 2001; Matsumoto et al., 2006).

There also is a potential for pathologies to interrelate with each other. For example, anemic hypoxia can develop in tumors due to the underlying systemic anemia of the patient (Vaupel & Mayer, 2014).

In addition to these underlying effects of pathology on tissue oxygen levels, any applied therapeutic interventions are very likely to induce changes. For example, cell killing due to radiation or chemotherapy will alter oxygen consumption patterns. These same therapies will also affect the O_2_ supplying vasculature via both antiangiogenic effects and—possibly—normalization of vessel structure (Jain, 2005). The effects of therapies will generally vary both spatially and temporally, reiterating the complexity of meaningfully characterizing tissue oxygen levels.

## 4 ANALYSIS OF THE ABILITY OF CLINICALLY AVAILABLE TECHNIQUES TO DIRECTLY MEASURE LEVELS OF O_2_ IN TISSUES AND/OR RESOLVE THE HETEROGENEITY OF O_2_ DISTRIBUTIONS IN TISSUES

Although many techniques are often thought to measure actual O_2_ in tissues, only a few actually have the potential to make O_2_ measurements directly in the tissues of interest (Springett & Swartz, 2007; Tatum et al., 2006). Techniques that can potentially assess O_2_ directly in tissues include: EPR (Epel et al., 2019; Swartz et al., 2014; Swartz et al., 2014), the Eppendorf electrode (Vaupel, Höckel, & Mayer, 2007), some optical methods based on direct measurements of target molecules in tissues, for example, phosphorescence quenching of optical sensors placed directly in tissues or as part of a physical probe such as the “OxyLite” (Wen et al., 2008), and NMR relaxation techniques (Colliez et al., 2014).

Two other types of measurements assess O_2_ in the vascular system. Blood gases do this directly, while optical methods that measure both hemoglobin saturation and total hemoglobin (especially near infrared spectroscopy [NIRS]; Scheeren, Schober, & Schwarte, 2012) provide a plausible link to the pO_2_ in the blood.

However, the techniques most often used clinically to characterize tissue oxygenation do not in fact measure O_2_ directly; instead, they measure “indirect” parameters that can be plausibly linked to actual O_2_ levels but only under appropriate/defined circumstances. This latter group of techniques includes positron emission tomography (PET) imaging of glucose derivatives (Neveu et al., 2015), PET imaging of drugs that localize in hypoxic tissues (Tran et al., 2012), laser Doppler flow, measures of metabolites that may be affected by O_2_ levels, for example, lactate and redox intermediates, and several magnetic resonance imaging/nuclear magnetic resonance (MRI/NMR), blood oxygenation level dependent (BOLD) imaging (Baudelet & Gallez, 2002), and MRI (Egeland et al., 2006). Note: If their basis is understood and the data considered accordingly, these can all provide clinically and physiologically useful information even though they do not provide direct information on the amount of O_2_ in the tissues.

### 4.1 Direct measures of O_2_ in targeted tissues that potentially can be used in human subjects

These are techniques that, while they have the capability of providing direct quantitative measurements of O_2_ in homogeneous media, cannot provide such data in tissues in vivo because the volumes that they sense are larger than the volumes of homogeneity of O_2_ in actively metabolizing tissues. Consequently, all in vivo measurements of O_2_ are inherently *averages* of the actual oxygen content in that volume. Even neglecting the need to include measures of heterogeneity *inside* cells, based on the usual volume of cells and assuming that differences are sought for aggregates of ≤3 cells, for a measurement of heterogeneity sensed within a 10 mm diameter volume, the spatial resolution needed to appropriately characterize O_2_ levels in this volume becomes 8 million voxels. The measuring techniques may not even provide a well‐defined averaged pO_2_ value within the volume that they sense. For example, sensors for the signal that is being measured.

In the next sections we review the characteristics of each technique that can directly measure O_2_ levels in tissues. We also briefly remark on the volumes they measure and how the measures obtained can be useful clinically (see also Ortez‐Prado, Dunn, Vasconez, Castillo, & Visco, 2019).

#### 4.1.1 EPR oximetry

Using appropriate particulate paramagnetic materials, EPR oximetry can provide direct measurements of O_2_, that is, the EPR signal is directly proportional to the amount of O_2_ (Epel, Bowman, Mailer, & Halpern, 2014; Swartz, Vaupel, et al., 2020). Because each multisite sensor senses a volume that is much larger than capillary networks, these techniques provide a volume averaged sampling of all compartments within the tissues. The time resolution of the techniques can be milliseconds or shorter.

The measured parameter of an EPR spectrum that indicates the amount of O_2_ present is the line width of the observed resonance peak. There usually is a fixed relationship between the line width and the amount of O_2_, with the relationship being specific for each type of paramagnetic material, for example, carbon, charcoal, or phthalocyanine particulates. Using particulate oximetric materials, measurements can be continuous over any span of time and can be repeated indefinitely (see example in Figure 2). The method requires that the sensing material be injected or implanted in one or more regions of interest, but thereafter all measurements can be made entirely noninvasively. Importantly the measurements can be carried out in a clinical setting and can fit into the workflow needed for patient care.

The initial clinical EPR measurements of oxygen in tissues have used India Ink as the oxygen sensor (Swartz et al., 2014). The carbon particles are the components that respond to oxygen (Lan, Beghein, Charlier, & Gallez, 2004). After injection of 30–50 µl of the suspension through a small needle, the carbon particles disperse nonuniformly through the local region as small extracellular aggregates. They are often engulfed by macrophages. The resulting EPR spectra in the region probed by the resonator (i.e., the surface coil used for signal detection) are a composite of the oxygen‐dependent line widths from each of the particles. In reality, because of the relatively broad lines from the India Ink particles, the range of “oxygen levels” that are likely to be present in the tissue, and the limited number of particles in each subregion, it is a challenge to resolve directly even the major groups of similar line widths. Therefore, using the observed line width to provide a quantitative measure of oxygen would seem to have modest utility in itself.

The other method of clinical EPR oximetry is based on the use of micro‐crystalline probes (e.g., LiPc, LiNc‐BuO), encapsulated in biocompatible polymers (Swartz et al., 2014). Clinical measurements currently are being performed using the “OxyChip” which consists of oxygen sensitive microcrystals of lithium octa‐n‐butoxynaphthalocyanine (LiNc‐BuO) embedded in polydimethylsiloxane (PDMS; Hou, Khan, Gohain, Kuppusamy, & Kuppusamy, 2018; Hou et al., 2016; Jarvis et al., 2016). The dimensions currently used in humans are cylinders that are 5 mm long with a diameter of 0.6 mm. The EPR signal from the sensor (OxyChip) reflects the pO_2_ within the PDMS, which itself reflects an average of the pO_2_ in contact with the external surface of the cylinder. The dimensions of the OxyChip are much greater than those of a microcirculatory unit and therefore reflect many different such units. The microcirculatory networks sampled by the OxyChip are likely to include regions with quite different O_2_ levels. The “values” of oxygen that are obtained therefore do not, in themselves, provide any information on the heterogeneity of the sampled capillary networks.

In contrast to EPR spectroscopy based on particulates with stable free radicals, EPR oximetry imaging can provide spatially resolved measurements using soluble paramagnetic materials with stable free radicals, especially nitroxides and trityls (Epel et al., 2014; Epel et al., 2019). An advantage of pulse techniques using spin lattice relaxation rate images of dissolved O_2_ spin probes is the virtual absence of confounding variation of the spin from the probe affecting the measurement of the effect of O_2_ on relaxation rates. This is of particular importance for low oxygenation levels where the spin probe concentration and its effect on the relaxation rate approaches that of O_2_ (Epel et al., 2019). Nonetheless, the volumes of even the smallest resolvable voxel in the images will still be too large to avoid averaging of the O_2_ levels sensed within each voxel. The time resolution is usually at least several minutes. This technique cannot provide repeated measurements without readministering the paramagnetic material each time. At this time, this technique is available only for preclinical use, because there are no FDA‐approved soluble paramagnetic materials.

#### 4.1.2 The Eppendorf electrode

The Eppendorf electrode has been used clinically to provide direct tissue pO_2_ measurements along electrode tracks. To create a track, a series of points is obtained by progressing the 200 µm microelectrode through the tissue in a sequence of “Pilgrim steps,” that is, the probe is advanced a prescribed distance and then withdrawn a fraction of that distance to minimize pressure effects. The volume sampled by each point is estimated to be 100–500 cells around the tip of the probe (Vaupel et al., 2007). Therefore, the measurement usually reflects the average of a range of pO_2_ values, especially in the presence of pathophysiology. The histogram of values of a tumor was thought to be a representative sample of three to seven tracks through the volume, although this still was only a sampling of the true heterogeneity of the tissue O_2_. Nevertheless, in tumors, very useful clinical correlations have been found with the number of points below a threshold value, for example, median pO_2_ or hypoxic fractions (Vaupel & Mayer, 2007). Note that this does not require individual, true absolute pO_2_ values. Instead, the separation between severely hypoxic and less hypoxic values relies on averaged oxygen tensions. When used with multiple point measurements (>70), this is a good but not infallible technique to determine the presence of hypoxic regions. Regardless of its potential advantages, unfortunately this technique is no longer available clinically. This technique also had some practical limitations, including difficulties to repeat the measurement in a given tissue subvolume because of the local trauma produced from the preceding measurements.

Currently the only version of the oxygen electrode suitable for use in humans is a technique requiring temporary implantation of an oxygen electrode that extends through the skin and skull and is used to monitor severe brain trauma. Such a device, while very useful for its intended purpose, is not suitable for measurements in tumors (Stewart et al., 2008).

#### 4.1.3 Optical methods

The OxyLite technology is based on quenching by O_2_ of fluorescence with a sensor whose diameter is 230–750 µm at the end of a fiber‐optic fiber (User Manual, 2020). This cross‐section corresponds to the diameter of a tissue subvolume of 10–75 mammalian cells. And, therefore, the sensor will provide an average of the distribution of pO_2_ values throughout this volume. The temporal resolution can be quite rapid. It could be used in a manner similar to the Eppendorf electrode to obtain a series of similarly averaged measurements at different locations.

Direct injection of phosphorescent agents can provide one way to directly sample tissue O_2_, where the signal comes from the phosphorescence lifetime changes that result from excited triplet state quenching of O_2_ (Wilson, Harrison, & Vinogradov, 2012). High resolution mapping with very fast time resolution can be provided, although care must be taken in choosing the probe that localizes in the compartments of interest, as some provide intracellular information, some provide purely extracellular information, and some are simply perivascular in nature (Esipova et al., 2011). A key part of advancing these molecular probes has been to ensure that they sample the oxygen in the environment with a buffer around them such that the oxygen diffuses into the sensor and any measurement of oxygen by the triplet state quenching does not alter the local oxygen level, making the signal potentially nonlinear. Many unprotected or bare nanoparticle probes can have a signal dependence which is not ideal for linearity with oxygen or repeated measurements, or they could have uncertain localization. Dendrimer particles of the Oxyphor complex have been used as biocompatible large particles with pegylation to the exterior to ensure a known biodistribution (Esipova et al., 2019). Their localization is largely extracellular as well, but of course the localization can be tailored by specifically altering the surface chemistry, and they have been used to create probes for many unique biological environments such as the gut, bone marrow, or brain.

One of the more promising methods for oxygen measurement in vivo has been through delayed phosphorescence from protoporphyrin IX (Mik et al., 2006), which is present in all tissues to some extent, produced in the mitochondria, and can be proactively enhanced by administration of aminolevulinic acid. This molecule has triplet state quenching by oxygen as well, and the reverse intersystem crossing that can occur in the absence of oxygen allows for a delayed fluorescence signal which is uniquely sensitive to the local oxygen environment. This work was pioneered in cardiac tissues by Mik (2013) and shown to be a logical way to sample tissue oxygen (Scholz, Cao, Gunn, Brůža, & Pogue, 2020).

An important aspect of all these optical methods is that the depth of measurement is dominated by the light input/output geometry, which is typically limited to just a millimeter or two of depth for diffuse illumination and detection. Microscopy studies are widespread as they lend themselves well to thin tissues and take advantage of coherence or optical confinement tools. Thicker tissues are less studied, as this regime is more dominated by the tissue scattering nature than the features of the light signals. The development of x‐ray induced Cherenkov‐excited luminescence methods (which concomitantly use a specially developed porphyrin Oxyphor PtG4, developed by David Wilson and Sergei Vinogradov, University of Pennsylvania) have been shown to be useful for imaging through a few centimeters of tissue. This takes advantage of the deeper penetration of x‐rays. It samples all emissions from the tissue, reconstructing the detectable light. The reconstruction then infers the site of the origin of the light where the radiation is absorbed and therefore which emits Cherenkov to the excited sensor molecule. This measurement therefore provides information on oxygen at that site (Pogue et al., 2018). This technique can provide excellent spatial resolution but more development of the technique is needed in order to take advantage of the benefits of spatial resolution. More generally, the benefits of optical methods come largely from their ability to tailor the imaging system to the problem. Currently, however, the logistics have not been solved for routine human use, nor have the probes been made in an FDA‐approved format for use in humans.

Photoacoustic tomography (PAT) is another optical technique that is becoming increasingly used to define perfusion and O_2_ saturation in preclinical cancer and investigational studies of breast tumors, with ongoing commercial development for broader clinical use in a number of ventures. The method relies on optical absorption of a pulsed laser source by chromophores in the tissue, including hemoglobin, to produce ultrasound waves which are detected at the surface. The major benefits of PAT in lesion imaging are high resolution for neovasculature imaging through ~1–2 cm of tissue, and the ability to image features of blood oxygen saturation with spectroscopic PAT. Quantitation of tissue oxygenation using PAT remains difficult because an indirect indicator of oxygenation is measured (i.e., blood oxygen saturation) and unknown tissue properties skew the light penetration in tissue, making accurate spectroscopic measurements challenging at nonsuperficial depths (Cox, Laufer, Beard, & Arridge, 2012).

#### 4.1.4 NMR relaxation methods

Based on the principle that molecular O_2_ impacts the relaxation time of nuclei (Bennett et al., 1987; Swartz et al., 1985), several different approaches have been developed to try to use the power of NMR to measure O_2_ in tissues (Matsumoto et al., 2006). The measurement of O_2_ by NMR using isotopes with spin (O^17^ for NMR; Zhu & Chen, 2011) which is measured when the O_2_ is incorporated into water (and hence may better be considered as a measure of metabolism (Gallez, Neveu, Danhier, & Jordan, 2017; Neveu et al., 2015) and O^15^ for PET (Hattori et al., 2004) is possible, but these are extraordinarily expensive and not suitable for routine clinical use.

The most developed method has been the use of the relaxation time of fluorine nuclei in fluorinated hydrocarbons injected directly into tissues (Liu et al., 2011; Zhao, Jiang, & Mason, 2004). The measurements are based on the relaxation time of the F atoms in the emulsion, but the volume of the fluorine containing hydrocarbon is much larger than several capillary networks. Consequently, the data obtained are an average of a range of partial pressures. To date this approach has not advanced to clinical use.

There are a number of other NMR‐related approaches that have been suggested using the impact of O_2_ on the relaxation of protons (Colliez et al., 2014; O'Connor et al., 2009, 2016). Potentially these might provide spatially resolved data that could be quite useful. However, it is very unlikely that any could have the resolution to resolve the spatial heterogeneity of O_2_ in tissues. Thus, these measurements of O_2_ will be similar to the other techniques discussed, that is, their data will be based on assessing changes in “averaged” O_2_ levels.

### 4.2 Measures of O_2_ in the vascular system

Measures of O_2_ in the vascular system are based on the known relationship between the O_2_ saturation of hemoglobin (Hb) and the ambient pO_2_ as shown in the sigmoidal oxygen dissociation curve. However, the vascular system is, by its nature, not uniform with respect to its saturation. Arterial and venous systems have very different saturations, with the capillaries (which is where most O_2_ is delivered to the tissue) being in between these extremes. Therefore, when measurements such as pulse oximetry or NIRS report HbO_2_ saturation (Sakata, Grinberg, Grinberg, Springett, & Swartz, 2005), it is important to understand what part of the vascular system is being measured.

The problem with applying measurements of oxygen made in blood to measurements in tissues is that, while the amount of O_2_ in the vascular system is an important parameter impacting O_2_ levels in the perfused tissues, it is not possible to go directly from measuring O_2_ in blood to knowing the O_2_ in tissues. That is because O_2_ in the tissue is determined by many factors in addition to the potential supply of O_2_ from the red blood cells. Perfusion and diffusion must occur to get into the tissues. In some tissues, such as skeletal muscle and myocardium, the situation is even more complicated because of binding of oxygen by myoglobin. For example, some consumption of oxygen by cells will occur between the vascular system and the tissues of interest, and the number of cells and level of oxygen consumption by the cells can vary greatly. If tumors are present, the situation is further impacted by the chaotic nature of the neovasculature that arises in the tumors, with poorer perfusion and abnormal diffusion out of the vessels (Collins, Rudenski, Gibson, Howard, & O'Driscoll, 2015).

The other type of measurement of O_2_ in the vascular system is the measurement of blood gases (Davis, Walsh, Sittig, & Restrepo, 2013). These are usually done in larger vessels. These measurements have the same limitations as NIRS in regard to being able to provide information on the O_2_ levels in the extravascular tissue compartment.

Even though they are not measuring the actual increase of oxygen in tissues, measurements in the vascular system may be helpful in assessing response to techniques to increase O_2_ availability, because of ease of use and potential depth of sampling (Kim & Liu, 2008; Sunar et al., 2006).

While these tools are volume averaging over centimeters of tissue and the signal is dominated by areas of higher perfusion, the data on changes can provide clinically valuable information indicating that the oxygenation technique could be effective in raising levels of oxygen in the tissues of interest.

A related methodology, which is widely available, BOLD MRI (Thulborn, Waterton, Matthews, & Radda, 1982), is sometimes mistakenly thought to provide a direct measurement of O_2_ in the vascular system. But because it provides only one parameter, the amount of DeoxyHb, it cannot be used to assess HbO_2_ saturation or pO_2_ in the circulatory system. In principle, changes in the amount of the BOLD signal could be used to provide an indication of changing O_2_ levels. However, this type of use is limited by the fact that, if the blood volume within the sampled voxels changes, the amount of DeoxyHb will change even if the saturation remains the same and, therefore, the results may be confounded (Baudelet & Gallez, 2002). The potential time resolution is very fast.

### 4.3 Other indirect measures of tissue oxygen

There are a number of methods used clinically that attempt to provide indications of O_2_ levels in tissues, which are based on plausible but indirect relationships to the actual O_2_ levels. While these therefore intrinsically do not directly measure O_2_ in tissues, because of their widespread availability and, perhaps, some naivety among clinical users, they are sometimes considered to provide direct measurements of O_2_. This confusion has important implications for their usefulness in clinical applications because, while these techniques may provide valid and quantitative measures of parameters associated with their biological or chemical modes of interaction, their quantitative relationships to O_2_ levels may be poorly defined and potentially misinterpreted.

The use of molecules such as the nitroimidazoles and Cu‐ATSM that selectively localize in hypoxic tissues (O_2_ content <1%) has been widely employed, most often using PET labels to indicate their location (Busk et al., 2020; Chapman, Franko & Sharplin, 1981; Gutfilen, Souza, & Valentini, 2018; Kelada *et al*., 2017; Tran et al., 2012). The principle is that with critically low levels of O_2_ these can be reduced to reactive intermediates which, if not reoxidized by O_2_, can then bind to cellular components (Sealy, Swartz, & Olive, 1978). While these probes are widely used and often have been clinically useful, they clearly cannot provide a direct measure of the O_2_ content of the tissue. They can, nonetheless, provide a qualitative indication of regions with moderate to good perfusion that were hypoxic at the time that the tracer was delivered. When the nature of the data is understood, these techniques can be quite useful.

Another widely used PET clinical imaging technique is based on the observation that many tumors have a high rate of anaerobic and aerobic glycolysis and therefore a high uptake of glucose, which in turn can be followed by using the imaging agent 18F‐FDG (18F‐fluorodeoxyglucose; Lopci et al., 2014). The amount of uptake is based on a number of different factors including perfusion rate, extent of hypoxia, expression of the Warburg effect (i.e., aerobic glycolysis), number of cells, uptake of glucose analogs and rate of glycolysis (Vaupel & Multhoff, 2020; Vaupel et al., 2019). Clinical use of this technique is widespread and considered clinically useful for a number of contexts, especially in identifying regions where metabolically active tumor cells are located. Some clinicians even consider areas of high uptake as potentially indicating tumor hypoxia although the Warburg effect is mainly responsible for the high glucose uptake rates (Vaupel & Multhoff, 2020). A major complication is the high rate of false positives, due to many other causes of uptake of the agent (Britton & Robinson, 2016; Metser & Even‐Sapir, 2007). There can be very significant discrepancies between the pattern of localization of FDG and the histological evidence of hypoxia (Christian et al., 2009).

### 4.4 Endogenous hypoxia markers and other surrogates

Endogenous hypoxia markers (e.g., HIF‐1α, GLUT‐1, CA IX) are often used to judge the oxygenation status of malignant tumors. Responses to low oxygen levels in normal and cancer cells are mainly initiated by HIF‐1α, a key regulator for genes responsible for mammalian oxygen homeostasis. These responses (among others) include an increased erythropoiesis, angiogenesis, and glycolysis. The latter is mainly initiated by an enhanced cellular glucose uptake through GLUT‐1 transporters (Semenza, 1999, 2000). However, the expression of these markers is also driven by a series of other parameters as described in Section 3.2. Therefore, these proteins per se are not useful at all to judge the oxygenation/hypoxia status of benign and malignant tumors. The respective problems have been discussed in detail by Mayer, Höckel, and Vaupel (2008).

There is also a group of physiological measurements that are sometimes indirectly linked to O_2_. Measurements of blood flow, for example, MRI perfusion (Essig et al., 2013) and laser Doppler flow (Briers, 2001) are often used clinically to obtain a (surrogate) parameter that is potentially linked to the supply of O_2_. Because of the many factors that affect how much O_2_ is delivered and the impact of utilization on the amount of O_2_ is available, these techniques cannot by themselves provide reliable insights into the oxygenation status of the tissues. Measurements of metabolites that may be affected by O_2_ levels, for example, lactate and redox intermediates, are frequently used. However, their levels are the result of complex interactions that depend on many factors in addition to the amount of available O_2_, and therefore cannot reliably indicate O_2_ levels in tissues. Nonetheless, these physiological measurements, especially if repeated and related to appropriate other parameters, may in some instances provide useful clinical information.

## 5 DISCUSSION

### 5.1 Clinical value of indirect measurements of oxygen in tissues

It seems clear from both fundamental principles and the discussion in the previous sections that the use of indirect methods to directly determine the values for oxygen levels in tissues is not logical. This does not mean that such measurements intrinsically have no clinical value, but such uses do require careful consideration of a number of factors. At a minimum it requires a separate link, or calibration, of those indirect measurements, for example, lactate levels, to a method that measures O_2_ directly in tissues. There also needs to be an understanding of other types of links between the indirect and direct methods, so that there is adequate consideration of all factors that can affect the validity of that link, for example, many factors in addition to hypoxia can increase lactate levels in tissues.

In one of very few studies that actually attempts a head‐to‐head comparison of indirect methods of assessing hypoxia in the same tissue in humans, Nordsmark et al. (2007) compared 4 “hypoxia‐specific” indirect assays in 67 patients with head and neck cancer with a direct measure of O_2_ (using the Eppendorf electrode). They found that the indirect techniques differed in their relationship to the O_2_ measured by the electrode, and there were few correlations among the indirect assays. It seems quite possible that, even with the indirect techniques that seemed to correlate with the direct method (i.e., high plasma osteopontin and high HIF‐1α correlated with the Eppendorf method), the likelihood is that indirect methods would not always correlate well with each other because these parameters can be impacted by other factors as well. However, if they are to be used to predict O_2_ levels, it is important to identify those that strongly correlate with direct measures and/or to understand what other factors need to be measured in order to relate the information to O_2_ levels.

Of course, linking different candidate methods to a validated direct method, whether they are directly or indirectly assessing O_2_ in tissues, can also be useful to confirm which candidate measures would actually be poor or unreliable indicators of O_2_ in deep tissues, so that they should not be used for that purpose. Transcutaneous pO_2_ or surface pulse oximetry are two such examples. These techniques are relatively easy and noninvasive methods that can be repeated; they have been used to monitor arterial O_2_ near the surface or to identify proximal ischemia in patients with peripheral vascular disease (Abraham et al., 2003) or to monitor neonates in critical care units (Salyer, 2003). Despite the potential validity of these other uses, they are unlikely to be validated for O_2_ in deep tissues, because there is no plausible quantitative link between measurements of O_2_ made at the *surface* of the skin and *deep* tissue O_2_ levels.

There are some clinical trials that are using indirect methods to make clinical decisions, which may, under appropriate conditions, provide very useful clinical information even if they are not directly measuring O_2_ in tissues. An excellent example is a recent study using the PET reagent fluoromisonidazole (FMISO) labeled with fluorine‐18. FMISO was used as a surrogate for tumor O_2_ to determine whether FMISO data can be used as a guide to de‐escalate the radiation dose in patients being concurrently treated with chemotherapy for oropharyngeal papilloma virus related advanced tumors (Lee et al., 2016).

The ultimate criterion for clinical utility of indirect methods to measure tissue O_2_ will be the impact on outcomes. If there appears to be a benefit from such measurements based on outcomes, it will, of course be important to delineate the circumstances where this information is valid. Validity should include a recognition that the basis of the findings are correlations of measures of O_2_ with outcomes are not based on rigorous measurements of O_2_. This recognition is needed to prevent inappropriate extension to other uses.

### 5.2 Clinical value of direct measurements of oxygen in tissues

As we have shown above, whatever measuring method is employed, the volume that is sensed by the method will always be too large to be truly homogeneous because of the variations of O_2_ levels across small distance in tissues; (the variations are on a submillimeter scale!). In addition, the amount of O_2_ in the interrogated volumes may change very quickly, for example, in cycling hypoxia. Consequently, even direct measurements of O_2_ in tissues cannot accurately be reported as being a true single value. Instead, all current in vivo measurements purported to be measures of O_2_ levels in tissues are reflecting complex combinations of O_2_ levels, that is, are truly based on a mélange, that is, a mix of pO_2_ or [O_2_] values.

It is important to recognize that use of the term “absolute” in specification of pO_2_ can foster misinterpretations, and, in turn, can lead to inappropriate applications of these data to both clinical decision‐making and basic science theory. This is because, while the term “absolute” is correctly used to describe gas partial pressure measured in defined units above the pressure of an ideal vacuum, the term can be misinterpreted to connote measurements that are perfect, unbounded, definite, certain, etc. and these meanings, as we have shown, are inaccurate and misleading when applied to the in vivo circumstance. At a minimum, attention should be applied when using and interpreting the term “absolute” to describe O_2_ measurements, especially in the in vivo setting, and usage should be restricted to quantitative specification of vacuum as the zero of the defined scale, as opposed to a descriptor of data quality.

Although the inaccuracy and possible clinical misinterpretation of current measures of O_2_ levels in vivo has been argued before (Vaupel & Mayer, 2017a), the practice of providing single discrete values as definitive descriptors of oxygen levels, often specified as “absolute pO_2_” has persisted in the in vivo setting. The practice is abetted by being correct in some circumstances, that is, the techniques, especially when applied in restricted laboratory conditions, appear to provide valid measurements of pO_2_ or other rigorous parameters within specific volumes or limited time domains. But even then a valid measurement of an absolute pO_2_ in tissue does not assure that pO_2_ accurately reflects the pO_2_ in the tissue, for example, an EPR measurement of macroscopic sensor accurately provides the average value within the sensor but that is unlikely to reflect the heterogeneity of the tissue around the sensor. Another factor that encourages misinterpretation is the availability of robust data from model homogeneous systems, for example, cell cultures, that appear to indicate that clinical outcomes should be related to tissue O_2_ when applied to in vivo situations as well. More specifically, in studies conducted using homogeneous cell suspensions where the O_2_ levels can be rigorously controlled, the relationship between radiation sensitivity of cells and pO_2_ can be clearly demonstrated in vitro, but not necessarily demonstrable in vivo.

While we argue that simple measurements of oxygen levels cannot fully characterize the heterogeneity present in tissues and can easily be misleading, we do not argue that current measures of O_2_ levels and the data the techniques can collect are without potential value. On the contrary, if the limitations of measurements of O_2_ are taken into account in using this information clinically, these measurements can be immensely useful clinically. It is well established that various techniques can be used to provide clinically useful information, such as clinically significant changes in O_2_ in tissues as a result of disease progression and/or treatment, or to detect the presence of hypoxia. These assessments may be quantitative (with the noted caveats on interpretation of numerical values) or qualitative in nature, where, for example, binary characterizations may be provided (e.g., increasing vs. decreasing). Moreover, detecting changes in O_2_ in response to hyperoxygenation can be used, especially in the presence of severe hypoxia, to identify those patients who might benefit from receiving “hyperoxygenation” during treatment or otherwise modifying treatment doses. These assessments can then be exploited to enhance diagnosis and therapy, while keeping in mind the caveats involved in the interpretations of the values obtained from the measurements.

There are a number of significant implications of the thesis presented here for physiological researchers as well as for clinicians. Already noted is the potential danger of making too simplistic inferences based on studies conducted in cell cultures (Carreau, El Hafny‐Rahbi, Matejuk, Grillon, & Kieda, 2011; Keeley & Mann, 2019). These problems arise because the O_2_ level can be rigorously controlled in cell cultures (in the bulk medium where O_2_ levels may be at air pressure, but not in the unstirred layer at the cell surface), but they cannot be controlled when applied to tumors in vivo.

The implications for using measures of O_2_ to identify hypoxia in tumors to target areas to receive increased radiation doses (referred to as “dose painting”) are less clear, but these measures too should be based on understanding what is being painted. Currently, discussions of the potential advantages of increasing dose to more hypoxic regions of tumors have often focused on acquiring and applying definitive measurements of “absolute” pO_2_. As argued in this paper, this goal is not only unobtainable but also unnecessary. The really crucial goal is to find where there are likely to be more hypoxic cells in the tumor and accurately portray these regions. At least in a preclinical model (Epel et al., 2019), it has been shown that these areas can be estimated, and these estimates use techniques that are potentially feasible to apply to clinical settings. Techniques that can be used to make repeated measurements of changes in O_2_ during the course of therapy would likely be especially valuable to determine if the treatments were leading to different relative changes in O_2_ levels.

The data in Figure 2 (which are presented as uncalibrated O_2_ sensitive EPR line widths) illustrate the potential clinical usefulness of using relative measurements of O_2_, that are not rooted in being “absolute” pO_2_ values. Recall that the data illustrate both the ability to gather repeated O_2_ measurements over a short time (minutes) and a long period (weeks or months). For clinical utility, having data from the short period (30 min in this case) could be used to determine whether a given patient's tumor is responsive to hyperoxic gas mixtures administered during preparation for and delivery of a typical fraction of radiation. This knowledge could help clinicians to determine when patients can benefit from strategies to enhance the effectiveness of radiation by using hyperoxygenation during treatment. The longer period of repetition (6 weeks in this case) could be used to monitor continued responsiveness of the tumor to hyperoxygenation during treatment and could monitor whether any changes occurred in the baseline measurements across a usual several‐week course of radiation.

As another example, there are considerable data from studies carried out 15–25 years ago using the Eppendorf histography, that is, using a polarographic system to make pretreatment assessments of the hypoxic fraction in patients and then, without changing therapy, looking at the relationships between pretreatment O_2_ data and outcomes (Höckel et al., 1993, 1996; Nordsmark et al., 2005).These findings, in brief, suggested a very strong prognostic relationship between identifying severely hypoxic tumors and having poorer outcomes following treatment.

These data were certainly “averaged” values of O_2_, in view of the intrinsic variability of such measurements (as illustrated by the multiple data acquisitions along several tracks in the Höckel/Vaupel laboratory), the variations among laboratories (Nordsmark et al., 2005), and the intrinsic nature of the data where each point inevitably includes data from a volume that is very likely to have heterogenous oxygen levels. In spite of these limitations and the fact that the measurements could not be repeated, it still could have been possible to apply their findings to change treatments based on their pretreatment measures. For example, patients with indications of high hypoxic fractions (i.e., Eppendorf measures suggested their tumor was very hypoxic) could have been prescribed treatments (assuming options were available) that were not as affected by the presence of large numbers of hypoxic cells (like avoiding radiation and instead using chemotherapy or immunotherapy). Alternatively, the patients could be targeted to utilize treatments designed to decrease the hypoxic fractions prior to or during treatment.

Although the Eppendorf histography system is no longer used in the clinic, its use resulted in very excellent insights into the importance of tumor hypoxia on the response to therapy. Especially if comparative measurements could be made repeatedly over time to follow treatment and attempts to modify local tissue oxygen that information could be used to personalize treatments, for example to deliver therapy under more favorable conditions and/or determine the effectiveness of interventions aimed at decreasing local hypoxia. In summary, a key implication, especially illustrated for cancer, is that the most useful clinical value of measurements of direct, or even indirect, measurements of O_2_ in tissue, is to follow temporal changes that occur by: (1) treatment‐enhancing interventions (especially hyperoxic interventions to increase O_2_ in the hypoxic regions of the tumor), (2) consequences of therapy (especially the impact from radiation but also immunotherapy, chemotherapy, hormone therapy, and surgery), and (3) disease progression. Of course, establishing the clinical value of these measurements of O_2_ will ultimately require appropriate studies of the outcomes of therapy using the O_2_ measurements to guide choices.

There are similar implications for the use of measurements of O_2_ in some other diseases. The most important other clinical application is likely to be for peripheral vascular disease (PVD). Relative measurements of O_2_ may help to identify and follow changes in levels of relatively low oxygenation (for arterial disease) or the location of tissues at risk for diabetic PVD (Editors, 2012). The clinically most import information may be to determine if there is a demonstrable impact on tissue O_2_ from interventions (drugs or surgical) and to follow the course of the disease.

For such applications the key information will be changes, not absolute values. So, the challenge is to have an ability to measure repeatedly at the same site with a parameter that reflects the oxygenation in the tissue. Such measurements have the potential to very significantly improve clinical outcomes, but, as noted for their use in cancer, the ultimate proof of their value needs to come from appropriate studies of outcomes.

Other types of clinical applications where changes rather than attempts to measure absolute values will be clinically valuable include: monitoring the status of healing wounds (including the status of tissue flaps, especially to determine the need for autologous skin transplants), and monitoring and avoiding untoward side effects of treatment (e.g., debilitating fibrosis caused by radiation or excessive chemotherapy‐induced peripheral nerve pain).

## 6 CONCLUSIONS

The bottom line is that clinical measurements of O_2_ in tissues will inevitably provide results that are, at best, based on data that do not fully reflect the inherent heterogeneity of O_2_ in tissues over space and time. Additionally, the nature of all existing techniques to measure O_2_ results in complex sampling of the volume that is sensed by the technique. By recognizing these potential limitations of the measures, one can focus on the very important and useful information that can be obtained from these techniques, especially data about factors that can change levels of O_2,_ and then exploit these changes diagnostically and therapeutically. Many examples already exist where measurements of oxygen in tissues have had significant clinical utility, even though in retrospect they really did not provide unambiguous information on absolute values of oxygen in the tissues. The clinical utility of such data ultimately needs to be verified by careful studies of outcomes related to the measured changes in levels of O_2_. For both basic physiological and clinical purposes, it is likely that the most useful information is likely to be obtained by employing multiple types of measurements that can thereby overcome the inevitable limitations of a single type of measurement.

## CONFLICTS OF INTEREST

HMS and ABF are owners of Clin‐EPR, LLC, which manufactures and sells clinical EPR instruments for investigational use only. HJH is a member of O2M Technologies which manufactures preclinical pO_2_ imagers and has pertinent patents.

## AUTHOR CONTRIBUTIONS

Drs. Swartz, Flood, and Vaupel are the lead authors. All authors have either contributed to the conception and design of this paper or to the interpretation of data and analyses as well as drafting or revising it critically for intellectual content. All have approved the final content of this work, agree to be accountable for all aspects of the work in ensuring that questions related to the accuracy or integrity of any part of the work are appropriately investigated and resolved, and represent all who qualify for authorship. We gratefully acknowledge all other scientists, clinicians, engineers, and coordinators on the PPG.
